# Incidence and Outcomes of COVID-19 Vaccine Hypersensitivity Reactions and Success of COVID-19 Vaccine Provocation Tests Post Previous COVID-19 Vaccine Hypersensitivity

**DOI:** 10.3390/medicines11060012

**Published:** 2024-05-27

**Authors:** Adi Kurniawan, Sukamto Koesnoe, Evy Yunihastuti, Hamzah Shatri

**Affiliations:** 1 Allergy and Immunology Subspecialty Education Programs, Department of Internal Medicine, Faculty of Medicine, University of Indonesia, Jakarta 10430, Indonesia; dr.adi.sppd@gmail.com; 2 Division of Allergy and Immunology, Department of Internal Medicine, Faculty of Medicine, University of Indonesia/Cipto Mangunkusumo Hospital, Jakarta 10430, Indonesia; 3 Division of Pyschosomatic and Palliative, Department of Internal Medicine, Faculty of Medicine, University of Indonesia/Cipto Mangunkusumo Hospital, Jakarta 10430, Indonesia; hshatri@yahoo.com

**Keywords:** hypersensitivity, COVID-19 vaccine, vaccination

## Abstract

**Background**: The COVID-19 pandemic has led to high mortality rates. There have been reports of hypersensitivity reactions with mild to severe symptoms. The COVID-19 vaccine provocation test is a vaccination protocol for individuals with a history of hypersensitivity. This study aims to determine the benefits of COVID-19 vaccine provocation tests in patients with a history of hypersensitivity reactions to COVID-19 vaccines and its influencing factors. **Objective**: To determine the incidence, severity, outcome of hypersensitivity reactions, and success of the COVID-19 vaccine provocation test. **Methods**: A retrospective cohort study was conducted, using subjects taken from medical record data at the RSCM who had received COVID-19 vaccination with a history of hypersensitivity. Data was taken from the COVID-19 vaccination records at the RSCM, BPJS Health Primary Care application. **Results**: From a total of 29,036 doses of the COVID-19 vaccine, 44 patients experienced hypersensitivity reactions. As many as 38.64% did not continue vaccination, 2.27% experienced mild hypersensitivity, and 59.44% were successfully vaccinated. **Conclusions**: People with a history of hypersensitivity reactions to COVID-19 vaccines can still receive subsequent COVID-19 vaccinations at healthcare facilities equipped with anaphylaxis kits and immunology allergists.

## 1. Introduction

The pandemic that was caused by the SARS-CoV-2 virus remains a global health problem. There is currently no effective treatment for SARS-CoV-2 infection, and vaccination is one of the effective strategies to end this pandemic by creating herd immunity within populations. Several COVID-19 vaccines have received global approval, including mRNA and viral vector vaccines. However, within 2 days of the first COVID-19 vaccination program held in the United States, there were two reports of severe hypersensitivity reactions to the COVID-19 vaccine. Understanding factors that can cause hypersensitivity reactions to the COVID-19 vaccines and developing prevention–management protocols for hypersensitivity reactions are crucial for the success of the vaccination program [1]. According to the Indonesian Ministry of Health, as of 25 November 2022, Indonesia has administered 205,409,201 first doses of the COVID-19 vaccine (87.53% coverage), 172,384,615 second doses (73.46% coverage), and only 66,474,137 third doses (28.33% coverage) [2,3,4,5,6,7].

Giving a vaccine gradually (graded challenge) or conducting a vaccine provocation test is an option to consider for patients with a history of severe hypersensitivity to the COVID-19 vaccine [8]. This approach is expected to reduce the risk of severe hypersensitivity reactions [9]. Given the limited data on COVID-19 vaccine provocation tests, this study aims to determine the benefits of COVID-19 vaccine provocation tests in patients with a history of hypersensitivity reactions to COVID-19 vaccines and its influencing factors.

## 2. Method

This study was a retrospective cohort study. The subjects were selected from the medical records at the Dr. Cipto Mangunkusumo Central General Hospital (RSCM) who had received COVID-19 vaccination with a history of hypersensitivity and/or anaphylaxis, with or without a provocation test. The study was conducted at the Dr. Cipto Mangunkusumo Hospital (RSCM) from January 2022 to November 2023. The population included vaccine recipients who underwent COVID-19 vaccination at the RSCM, and the accessible population consisted of vaccine recipients who experienced hypersensitivity reactions to the COVID-19 vaccine at the Dr. Cipto Mangunkusumo Hospital. The research sample included vaccine recipients with a history of hypersensitivity and/or anaphylaxis who underwent a provocation test or not at the Dr. Cipto Mangunkusumo National General Hospital and who met the inclusion and exclusion criteria. The inclusion criteria were participants who were vaccinated at the RSCM, and the exclusion criteria were hypersensitive reactions not reported at the RSCM.

Data was collected through COVID-19 vaccination records that were held at the RSCM. The data on vaccine recipients at the RSCM were obtained from the database in the Primary Care division of the Social Health Insurance Administration Body (BPJS Kesehatan), which was filled out by RSCM administrative staff. However, some vaccine recipients were not registered in the application, so their basic information was recorded manually. This study involved 44 events, and further analysis was conducted to obtain the proportion of each variable. The basic characteristics of the research subjects were presented in the form of frequency tables. This study has obtained approval from the Medical Research Ethics Committee of the FKUI—RSCM: ND-558/UN2.F1/ETIK/PPM.00.02/2023.

## 3. Result

**Figure 1:**
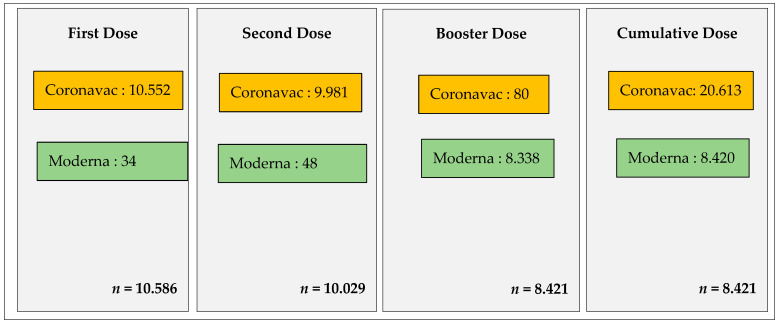
Type of COVID-19 vaccines usd during first, second, boosters and cumulative dose periods.

This study involved 29,036 participants who underwent COVID-19 vaccination with doses 1, 2, and booster 1 at the RSCM. During the first dose vaccination period, 10,586 doses were administered, with 10,029 doses administered during the second dose period, and 8421 doses were given during the first booster period. The most widely administered type of COVID-19 vaccine was the inactive vaccine, Coronavac, accounting for 20,613 doses (70.99%), followed by mRNA vaccines, including 8420 doses of Moderna (28.99%) and 3 doses of Pfizer (0.01%) (Figure 1). 

In this study, from the total number of doses of the COVID-19 vaccine that were given to the participants for doses 1, 2, and the first booster, it was found that 44 participants receiving the COVID-19 vaccine experienced hypersensitivity reactions (0.15%). Most hypersensitivity reactions occurred in the female gender group, with 35 participants (79.5%), while 9 individuals (20.5%) were male. Age characteristics were dominated by those below 50 years of age, with 37 individuals (84.1%), and the group above 50 years of age had 7 individuals (15.9%).

A total of 20,613 doses of the Coronavac vaccine were administered during the COVID-19 vaccination across the first dose, second dose, and first booster periods. It was reported that 20 participants (0.09%) receiving the Coronavac vaccine experienced hypersensitivity reactions. The first dose period had the highest number of hypersensitivity reactions (*n* = 13), and the second dose period had 7 cases. In contrast, no hypersensitivity reactions were found during the first booster period during Coronavac vaccination.

A total of 8420 doses of the Moderna vaccine were administered during the vaccination periods for doses 1, 2, and the first booster. The total occurrence of hypersensitivity reactions to the Moderna vaccine was 0.309%. The period with the highest occurrence of Moderna vaccine hypersensitivity reactions was during the first booster dose (*n* = 23), with just 1 case in the first dose period. In contrast, no hypersensitivity reactions were found during the second dose period. No reports of hypersensitivity reactions to the Pfizer vaccine were found. However, the assessment of hypersensitivity reaction incidents to the Pfizer vaccine could not be evaluated because the number of doses given was not the same as the number of doses of other COVID-19 vaccines. The incidence of hypersensitivity reactions in vaccine recipients at the RSCM using mRNA vaccines (*n* = 24) was higher than that with the Coronavac vaccine (*n* = 20).

**Figure 2:**
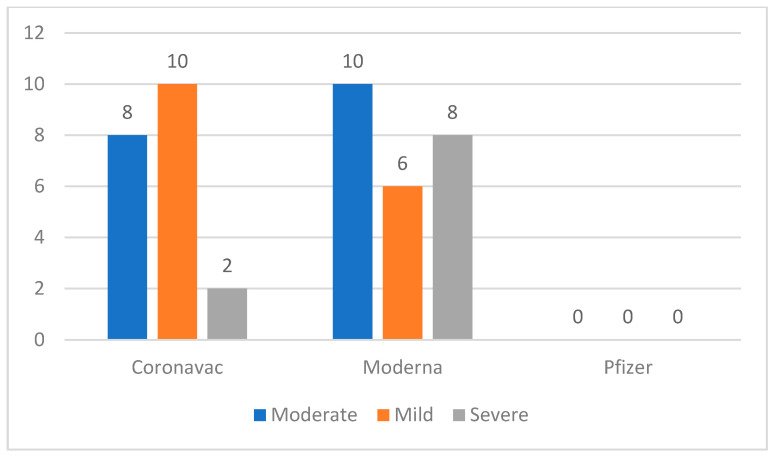
Proportion of hypersensitivity reactions based on the degree of hypersensitivity to the COVID-19 vaccine.

Out of the 44 COVID-19 vaccine recipients who experienced hypersensitivity reactions, the Moderna vaccine group had the highest number of hypersensitivity reactions, with 33.33% classified as severe, 25% as moderate, and 41.67% as mild. For the Coronavac vaccine, the majority were classified as moderate (50%), followed by mild (40%) and severe (10%) (Figure 2).

**Figure 3:**
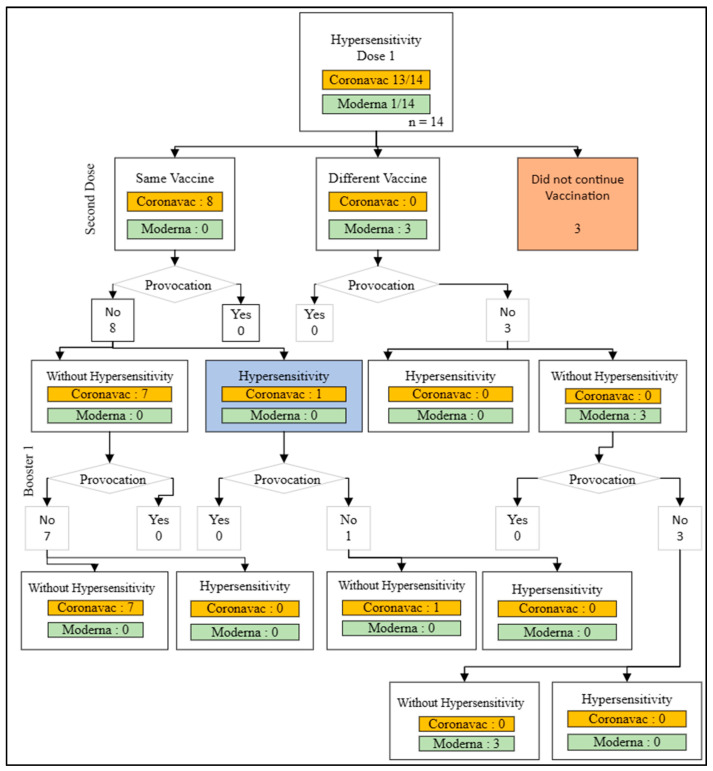
Action flow after dose 1 hypersensitivity reaction.

In the first dose, there were 13 cases of hypersensitivity reactions to the COVID-19 vaccine, with 12 cases of the Coronavac vaccine and 1 case of the Moderna vaccine. The participants who experienced hypersensitivity reactions to the COVID-19 vaccine in the first dose (13 individuals) were divided into three groups: continuing vaccination with the same vaccine (8 individuals), switching to a different vaccine (2 individuals), and a group that did not continue vaccination (3 individuals). (Figure 3). 

In the two remaining groups, whether using the same vaccine or a different one, vaccination for the second dose proceeded without conducting a provocation test. One of the 8 people who received the identical vaccination suffered hypersensitive responses, whereas the other seven did not. The first booster dosage was administered without a provocation test, and none of the eight vaccination recipients exhibited hypersensitivity responses after receiving the first booster dose.

**Figure 4:**
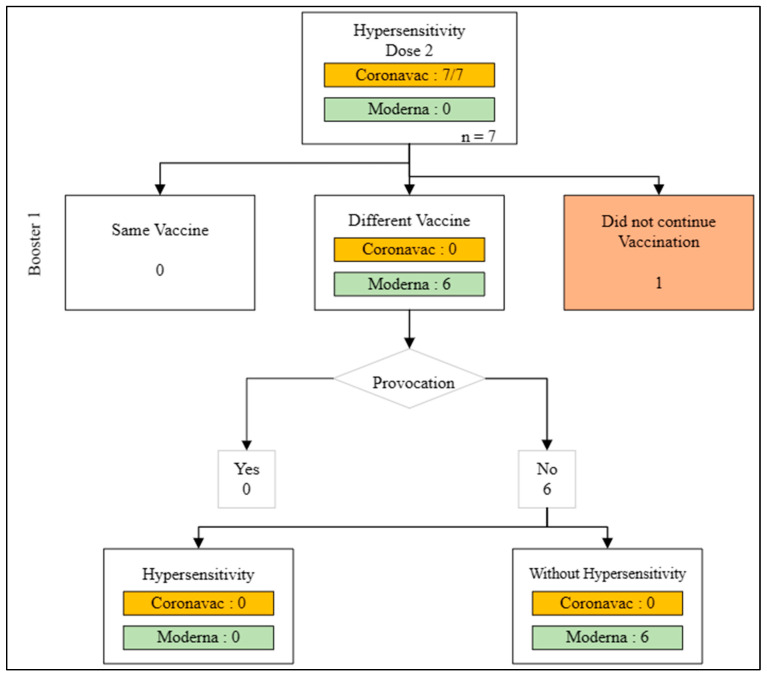
Action flow after dose 2 hypersensitivity reaction.

In the second dose of COVID-19 vaccination, a total of 10,029 doses were administered. The majority of these doses were the Coronavac vaccine, with 9981 doses (99.52%), and the Moderna vaccine, with 48 doses (0.47%). There were 7 incidences of hypersensitivity responses to the vaccination, all of which were linked to the Coronavac vaccine. One participant declined further immunization, while the remaining 6 people received the COVID-19 booster 1 vaccination using a different vaccine without a provocation test. There were no hypersensitivity events in these 6 people after the first booster dosage (Figure 4).

Unlike the first and second doses, the third dose, also known as booster 1, experienced a decrease in the number of doses given to the recipients, totaling 8421 doses. The Moderna vaccine was the most administered during this period, with 8338 doses (99.01%), followed by 80 doses of Coronavac (0.95%). Hypersensitivity reactions were found in 23 doses of Moderna during booster 1. This group was divided into three categories: 13 individuals who chose not to continue COVID-19 vaccination, 9 individuals who continued vaccination with a different vaccine, and one individual who continued with the same vaccine.

**Figure 5:**
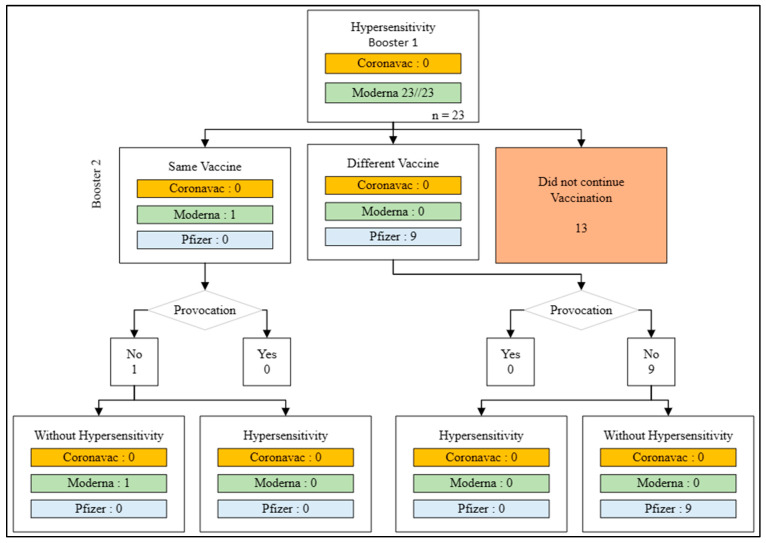
Action flow after hypersensitivity reaction to the first booster.

In the group receiving a different vaccine for the second booster dose, vaccination was conducted without a provocation test, and no hypersensitivity reactions were found after vaccination. Similarly, in the group of healthcare workers receiving the same vaccine for the second booster dose, without a provocation test, no hypersensitivity reactions were observed (Figure 5). 

Hypersensitivity reactions to the COVID-19 vaccine led to 17 recipients of the COVID-19 vaccine choosing not to continue with the subsequent vaccine doses.

In this study, a total of 25 patients underwent a provocation test for the COVID-19 vaccine. The characteristics of these patients undergoing the COVID-19 vaccine provocation test shared some similarities with the group of vaccine recipients who experienced hypersensitivity reactions to the COVID-19 vaccine but proceeded with the next vaccine doses without undergoing a provocation test (Table 1).

Among the patients, 17 (68%) had obesity, while 8 (32%) were non-obese. Based on the severity of hypersensitivity reactions, the majority were classified as grade 1, with 10 patients (40%), followed by grade 2 with 7 patients (28%), and grade 3 with 6 patients (24%). The majority of those experiencing hypersensitivity reactions were female, with 17 patients (68%), while males accounted for 8 patients (32%). The age group under 50 years old had the highest number of hypersensitivity reactions, with 16 patients (64%), while the age group over 50 years old had 9 patients (36%). Patients with a history of comorbidities were not significantly different from those without comorbidities, comprising 13 patients (52%) and 12 patients (48%), respectively.

The most frequently used vaccine was Sinovac, administered to 10 patients (40%), followed by Pfizer with 7 patients (28%), Moderna with 6 patients (24%), and AstraZeneca with 2 patients (8%). Comorbidities were present in 7 patients, including asthma in 5 patients (42%) and one case each of pulmonary tuberculosis, dermatitis, systemic lupus erythematosus (SLE), vasculitis, urticaria, and cardiovascular diseases (8% each). A history of drug hypersensitivity was reported in 8 patients (58%), food hypersensitivity in 2 patients (14%), and both drug and food hypersensitivity in 4 patients (28%). Skin prick tests yielded positive results in 2 patients (8%) and negative results in 23 patients (92%). In the examination of total IgE levels, 12 patients (48%) had total IgE levels below the normal value of 87, while 13 patients (52%) had levels above 87. The success rate of the provocation test reached 92%, with 8% considered unsuccessful.

Among the 25 patients who underwent the vaccine provocation test in this study, 2 patients (8%) experienced hypersensitivity reactions during the test. As a result, the vaccine provocation test procedure was discontinued (deemed unsuccessful) for these 2 patients, while 23 patients (92%) successfully underwent the vaccine provocation test without any hypersensitivity reactions. Patient number 3 experienced an immediate hypersensitivity reaction with initial symptoms of a widespread red rash, shortness of breath, and decreased consciousness during the Sinovac Skin Prick Test. Epinephrine was injected intramuscularly, and the patient regained consciousness. Vital signs stabilized, the provocation test was stopped, and the patient was observed in the inpatient ward.

## 4. Discussion

There were 29,036 first, second, and first booster doses of the COVID-19 vaccine administered to the vaccination participants at the RSCM from July 2021 to November 2022. In the first dose vaccination, a total of 10,586 doses were given, consisting of 10,552 doses of the Coronavac vaccine and 34 doses of the Moderna vaccine. The second dose was still dominated by the Coronavac vaccine with 9981 doses. However, a shift in the types of vaccines occurred during the first booster dose period, with Moderna being the most frequently administered at 8338 doses, followed by 80 doses of Coronavac and 3 doses of Pfizer. There was a decreasing trend in COVID-19 vaccination coverage at the RSCM, consistent with national data showing a decline in vaccine coverage during each period.

A decrease in COVID-19 vaccine coverage was also observed in Brazil. Initially, the first dose coverage for the COVID-19 vaccine reached 80% for the age group 60–69 years, increasing to 95% for the age group above 70 years. However, during subsequent doses, there was a significant and drastic decrease, with the coverage for COVID-19 vaccination only reaching 26% [10].

There were several factors that contributed to the decline in COVID-19 vaccine doses, namely safety concerns, vaccine protection levels against the virus, fears and risks of side effects, lack of knowledge about the vaccine, lack of awareness about virus infection, misinformation about the vaccine and the virus, doubts about the efficacy of the vaccine, myths about vaccine side effects from anti-vaccine groups, and government vaccination program policies. Overall, these factors led to a decrease in COVID-19 vaccination coverage [11]. This study explored hypersensitivity reactions to the COVID-19 vaccine in RSCM participants, which could be one of the factors contributing to the decline in COVID-19 vaccination.

The study found that 44 vaccinated participants experienced hypersensitivity reactions during the first, second, and first booster doses. The majority were females (79.5%) compared to males (20.5%), consistent with studies in other countries.

The mechanism underlying the greater prevalence of hypersensitivity responses to the COVID-19 vaccination in females remains unknown. One hypothesis is that females have a higher initial immunological response to antigens than men due to regulation of the innate immune system. Generally, females exhibit high expression of IFN I, innate immune response, and T cell-related genes. Differences in immune response related to gender are influenced by genes on the X chromosome and polymorphisms in the ChrY gene regulated by epigenetic mechanisms. Additionally, hormonal factors play a role, with varying levels of sex hormones at different stages of life that affect immune cells. It is known that testosterone has a suppressing effect on both innate and adaptive immune responses, suggesting that sex hormones may play a role in increasing vaccine reactogenicity in females [12].

Genetic factors can also play a role in vaccine reactogenicity through interaction with sex hormones. The ACE2 receptor and Ang-II type 2 gene, both located on the X chromosome, can increase the immune response in females and raise the risk of vaccine-related side effects. Females have a higher risk of diseases related to mast cells, such as anaphylaxis, than males. The study concludes that hypersensitivity reactions to the COVID-19 vaccine consistently have higher rates in the female gender, suggesting considerations for different vaccine doses based on gender [12].

This study found that hypersensitivity reactions to the COVID-19 vaccine were more prevalent in the age group below 50 years (84.1%) compared to the age group above 50 years (15.9%). In a study by Beatty et al. in 2021 involving 19,586 patients with hypersensitivity reactions to the COVID-19 vaccine, the median age of the analyzed age group was 54 years [13]. Another study by Sainan Bian et al. in 2022 in China divided hypersensitivity reactions to the COVID-19 vaccine into two groups: anaphylaxis reactions and non-anaphylaxis reactions. In the anaphylaxis group, the median age was 45 years, and in the non-anaphylaxis group, the median age was 47 years [14].

The severity of hypersensitivity reactions varied, ranging from mild to severe. This study classified the severity into three groups based on systemic symptoms involving the respiratory and cardiovascular systems. The results revealed that the group with mild severity was the largest, comprising 18 vaccine recipients (41%), followed by the group with moderate severity, comprising 16 participants (36%), and the group with severe severity, comprising 10 participants (23%) [15].

This result disagrees with the findings of Trine et al. in Denmark 2021. The team evaluated individuals with a history of hypersensitivity responses to the COVID-19 vaccine and discovered that further vaccinations could be undertaken safely following diagnostic testing. Trine et al. reported 55 patients with hypersensitivity reactions who underwent a skin prick test (SPT), 52 of which were able to receive the next vaccine dose without experiencing hypersensitivity reactions. However, three patients tested positive, with one being allergic to Polyethylene Glycol and the others to 1,2-Dimyristoyl-rac-glycero-3-methoxypolyethylene glycol-2000. Subsequently, all three patients successfully received the next vaccine dose. Trine et al. concluded that with adequate diagnostic testing, patients with a history of hypersensitivity reactions to the COVID-19 vaccine can safely undergo subsequent vaccination without experiencing rapid hypersensitivity reactions [16]. In 2021, Benerji et al. provided recommendations for approaching patients with a history of hypersensitivity reactions to the COVID-19 vaccine. If there is a history of anaphylaxis suspected to be caused by allergens such as Polysorbate 80, Polyethylene Glycol injection, vaccines containing Polyethylene Glycol, or oral Polyethylene Glycol, the next vaccine dose can be administered directly with extended post-vaccination observation. However, for patients with a history of hypersensitivity reactions to Polyethylene Glycol and Polysorbate 80, it is recommended to conduct a skin prick test before vaccination [17].

In the group of patients who underwent the COVID-19 vaccine provocation test, examinations of immunoglobulin E (IgE) levels were also conducted. Sixty percent of patients had IgE levels above the reference value (>87), while 40% of patients had normal immunoglobulin levels. A study conducted by Mariko et al. in 2022 also examined IgE levels with more specific IgE levels, which is IgE from Polyethylene Glycol in patients with a history of hypersensitivity reactions to the COVID-19 vaccine. The discussion revealed the role of vaccine excipients, especially Polyethylene Glycol, in causing hypersensitivity reactions, particularly in mRNA vaccines. Although several studies have explored this topic, the mechanisms of hypersensitivity reactions to the COVID-19 vaccine are not fully understood [18].

This research is the first research on hypersensitivity reactions to the COVID-19 vaccine in Indonesia, which provides an overview of the incidence, severity, and outcome of hypersensitivity reactions. This research also carried out a COVID-19 vaccine provocation test, which is an action that has not been widely performed in allergy immunology health service centers in Indonesia. In this study, the total number of doses administered was quite large, namely 29,061, divided into doses 1, 2, and booster 1. However, the distribution of vaccine types varied, with some types administered in quite large numbers. In contrast, other types of vaccine were very few. Consequently, for certain COVID-19 vaccines, the occurrence of hypersensitivity reactions cannot be assessed due to the small number of doses administered.

## 5. Conclusions

The severity of hypersensitivity reactions to the COVID-19 vaccine recipients was predominantly in the mild category. Seventeen vaccine recipients of the COVID-19 vaccine did not proceed with the next dose after experiencing hypersensitivity reactions. In comparison, 27 vaccine recipients who had previously experienced hypersensitivity reactions successfully received the next vaccine dose without any hypersensitivity reaction. The success rate of the COVID-19 vaccine provocation test in patients with a history of previous hypersensitivity reactions to the COVID-19 vaccine was high.

## 6. Recommendation

A history of hypersensitive responses to prior doses of the COVID-19 vaccine may allow an individual to receive the COVID-19 vaccination. A bigger sample size is required for future studies to examine the association between the success rate of vaccination provocation testing and those who have suffered hypersensitivity responses.

## Figures and Tables

**Table 1:** Characteristics of patients who experienced hypersensitivity reactions to the COVID-19 vaccine and underwent a COVID-19 vaccine provocation test for the next dose.

Variable	Vaccinated Patients (*n* = 25)	
	*n*	%
Age		
<50 years	16	64
≥50 years	9	36
Gender		
Woman	17	68
Man	8	32
Obese		
Yes	17	68
No	8	32
Vaccine Type		
Sinovac	10	40
Pfizer	7	28
Moderna	6	24
Comorbid		
Asthma	5	42
Pulmonary Tuberculosis	1	8
Dermatitis	1	8
SLE	1	8
Vasculitis	1	8
Urticaria	1	8
Cardiovascular	1	8
Hypersensitivity History		
Drug	8	58
Food	2	14
Drug and Food	4	28
Skin Prick Test		
Positive	2	8
Negative	23	92
Total IgE		
<87	12	48
>87	13	52
Successful Provocation		
Yes	23	92
No	2	8

## Data Availability

The raw data supporting the conclusions of this article will be made available by the authors on request.

## References

[1] Romantowski J., Kruszewski J., Solarski O., Bant A., Chciałowski A., Pietrzyk I., Sańpruch P., Górska A., Chełmińska M., Knurowska A. (2022). Protocol of safe vaccination against COVID-19 in patients with high risk of allergic reactions. Clin. Transl. Allergy.

[2] Kemenkes R.I. Cakupan Vaksinasi COVID-19 di Indonesia. https://layanandata.kemkes.go.id/katalog-data/covid-19/ketersediaan-data/vaksinasi-covid-19.

[3] Amarasinghe A., World Health Organization, Global Advisory Committee on Vaccine Safety (2014). Global Manual on Surveillance of Adverse Events Following Immunization.

[4] Baden L.R., El Sahly H.M., Essink B., Kotloff K., Frey S., Novak R., Diemert D., Spector S.A., Rouphael N., Creech C.B. (2021). Efficacy and Safety of the mRNA-1273 SARS-CoV-2 Vaccine. N. Engl. J. Med..

[5] Polack F.P., Thomas S.J., Kitchin N., Absalon J., Gurtman A., Lockhart S., Perez J.L., Pérez Marc G., Moreira E.D., Zerbini C. (2020). Safety and Efficacy of the BNT162b2 mRNA Covid-19 Vaccine. N. Engl. J. Med..

[6] Voysey M., Clemens S.A.C., Madhi S.A., Weckx L.Y., Folegatti P.M., Aley P.K., Angus B., Baillie V.L., Barnabas S.L., Bhorat Q.E. (2021). Safety and efficacy of the ChAdOx1 nCoV-19 vaccine (AZD1222) against SARS-CoV-2: An interim analysis of four randomised controlled trials in Brazil, South Africa, and the UK. Lancet.

[7] Tanriover M.D., Doğanay H.L., Akova M., Güner H.R., Azap A., Akhan S., Köse Ş., Erdinç F.Ş., Akalın E.H., Tabak Ö.F. (2021). Efficacy and safety of an inactivated whole-virion SARS-CoV-2 vaccine (CoronaVac): Interim results of a double-blind, randomised, placebo-controlled, phase 3 trial in Turkey. Lancet.

[8] Nilsson L., Csuth Á., Storsaeter J., Garvey L.H., Jenmalm M.C. (2021). Vaccine allergy: Evidence to consider for COVID-19 vaccines. Curr. Opin. Allergy Clin. Immunol..

[9] Leek T.K.V., Chan E.S., Connors L., Derfalvi B., Ellis A.K., Upton J.E.M., Abrams E.M. (2021). COVID-19 vaccine testing & administration guidance for allergists/immunologists from the Canadian Society of Allergy and Clinical Immunology (CSACI). Allergy Asthma Clin. Immunol..

[10] Kupek E. (2021). Low COVID-19 vaccination coverage and high COVID-19 mortality rates in Brazilian elderly. Rev. Bras. Epidemiol..

[11] Dhama K., Sharun K., Tiwari R., Dhawan M., Bin Emran T., Rabaan A.A., Alhumaid S. (2021). COVID-19 vaccine hesitancy–reasons and solutions to achieve a successful global vaccination campaign to tackle the ongoing pandemic. Hum. Vaccines Immunother..

[12] Green M.S., Peer V., Magid A., Hagani N., Anis E., Nitzan D. (2022). Gender Differences in Adverse Events Following the Pfizer-BioNTech COVID-19 Vaccine. Vaccines.

[13] Beatty A.L., Peyser N.D., Butcher X.E., Cocohoba J.M., Lin F., Olgin J.E., Pletcher M.J., Marcus G.M. (2021). Analysis of COVID-19 Vaccine Type and Adverse Effects Following Vaccination. JAMA Netw. Open.

[14] Bian S., Li L., Wang Z., Cui L., Xu Y., Guan K., Zhao B. (2022). Allergic Reactions After the Administration of COVID-19 Vaccines. Front. Public Health.

[15] Nelson E.J., McKune S.L., Ryan K.A., Lednicky J.A., Crowe S.R., Myers P.D., Morris J.G. (2021). SARS-CoV-2 Positivity on or after 9 Days among Quarantined Student Contacts of Confirmed Cases. JAMA J. Am. Med. Assoc..

[16] Rasmussen T.H., Mortz C.G., Georgsen T.K., Rasmussen H.M., Kjaer H.F., Bindslev-Jensen C. (2021). Patients with suspected allergic reactions to COVID-19 vaccines can be safely revaccinated after diagnostic work-up. Clin. Transl. Allergy.

[17] Banerji A., Wolfson A.R., Wickner P.G., Cogan A.S., McMahon A.E., Saff R., Robinson L.B., Phillips E., Blumenthal K.G. (2021). COVID-19 Vaccination in Patients with Reported Allergic Reactions: Updated Evidence and Suggested Approach. J. Allergy Clin. Immunol. Pract..

[18] Mouri M., Imamura M., Suzuki S., Kawasaki T., Ishizaki Y., Sakurai K., Nagafuchi H., Matsumura N., Uchida M., Ando T. (2022). Serum polyethylene glycol-specific IgE and IgG in patients with hypersensitivity to COVID-19 mRNA vaccines. Allergol. Int..

